# Measurement issues in longitudinal studies of mental health problems in children with neurodevelopmental disorders

**DOI:** 10.1186/s40359-025-02450-4

**Published:** 2025-03-18

**Authors:** Magnus Ivarsson, Henrik Danielsson, Christine Imms

**Affiliations:** 1 Department of Behavioural Sciences and Learning, Linköping University, Linköping, Sweden; 2 Department of Paediatrics and Healthy Trajectories Child and Youth Disability Research Hub, University of Melbourne, Melbourne, Australia; 3 Murdoch Children’s Research Institute, Melbourne, Australia

**Keywords:** Mental health problems, Child, Adolescent, Neurodevelopmental disorders, Longitudinal studies, Surveys and questionnaires, Bias

## Abstract

**Purpose:**

To develop and test an approach for assessing the risk of bias in four measurement-related domains key to the study of mental health problem trajectories in children with neurodevelopmental disorders (NDD): (1) conceptual overlap between mental health problems and NDD diagnostic criteria, (2) over-reliance on a single informant, (3) unwarranted omission of the child’s perspective, and (4) the use of instruments not designed for or adapted to the population.

**Methods:**

Building upon a previous systematic review, this study established supplementary criteria for assessing the risk of bias domains. Following this, the criteria were applied to measures used in 49 longitudinal studies of mental health problems in children with NDD.

**Results:**

The general risk of bias across domains was rated as high in 57.1% of the 49 included studies. The highest risk of bias was seen in domain four (rated as high in 87.8% of studies) and the lowest in domain three (24.5%).

**Conclusions:**

The risk of bias items enhance our understanding of the quality of the evidence about mental health problem trajectories in children with NDD. The methodological quality of future research can be increased by selecting conceptually clear scales developed for the population - preferably in the form of cognitively accessible self-report scales - and adopting a multi-informant approach.

**Supplementary Information:**

The online version contains supplementary material available at 10.1186/s40359-025-02450-4.

## Introduction

Neurodevelopmental disorders (NDD) such as intellectual disability (ID), autism spectrum disorder (ASD), and cerebral palsy (CP) have repeatedly been linked to heightened levels of mental health problems and mental disorders across childhood [1–6]. However, the measurement of longitudinal trajectories of mental health problems in children with NDD is associated with specific methodological challenges, relating to the interplay among the longitudinal design, study group characteristics, and the standard methods for measuring mental health problems in children. In a recent systematic review of longitudinal mental health problem trajectories in children with NDD [7], we observed that the instruments and checklists used to assess the risk of bias [8] were inadequate in addressing some of these challenges. Specifically, four domains related to aspects of outcome measurement stood out: (1) conceptual overlap between mental health problems and NDD diagnostic criteria, (2) over-reliance on a single informant, (3) unwarranted omission of the child’s perspective, and (4) the use of instruments not designed for or adapted to the population. The assessment of these domains, in our review, required more detailed data to be extracted from the included studies, and additional consideration of risks of bias. The present study aimed to develop an approach for evaluating the risk of bias in the four domains and to investigate the extent to which the four domains influence the validity of the findings about longitudinal trajectories of mental health problems in children with NDD.

The first of these specific risks concerns the potential conceptual overlap between mental health problems and NDD constructs [9]. It stems from the fact that mental illness, mental health problems, and mental disorders may overlap, depending on how they are defined [9]. In the Diagnostic and Statistical Manual of Mental Disorders (5th ed.; DSM-5 [10]), a mental disorder is defined as a syndrome “characterized by clinically significant disturbance in an individual’s cognition, emotion regulation, or behavior that reflects a dysfunction in the psychological, biological, or development processes underlying mental functioning” (p. 20). According to this definition, mental disorders encompass conditions that are typically regarded as mental illnesses (i.e., anxiety disorders and major depressive disorder), NDD (i.e., disorders typically manifested early in development, characterised by developmental deficits that produce impairments of personal, social, academic, or occupational functioning [10]), and other diagnoses. Mental health problems are commonly regarded as conceptually similar to mental illness, but as a broader construct, also covering milder problems and distress not meeting the criteria for a mental disorder [9]. In the present review, both internalising – (e.g., depression and anxiety) and externalising problems (e.g., aggressive behaviour) were considered as part of the mental health problem umbrella. Although this meant further widening of the mental health problems concept, it was necessary to enable comparisons of different aspects of emotional and behavioural problems. We also adopted a broad understanding of NDD by including childhood sensory and motor disorders, brain injuries acquired in childhood, and other diagnoses associated with sensory, motor, and mental impairments, in addition to the diagnoses listed as NDD in the DSM-5 [10] or the ICD-11 [11]. Many diagnoses involving such impairments that are not listed as NDD in the DSM-5 [10] arguably share important characteristics with those that are. For example, CP [12] and childhood hearing loss [13] are also characterised by developmental deficits that may produce impairments in different aspects of functioning. Moreover, conditions such as spina bifida [14] and CP [15] have previously been described as NDDs, with the latter showing genetic overlap with other NDDs, including intellectual disability and autism.

Importantly, an overlap between the mental health problem studied and the NDD is not problematic per se, but it may be, depending on the study’s aim and the interpretation of results. There is substantial symptomatic overlap across the mental disorders listed in the DSM-5 [16]. For example, attention deficit hyperactivity disorder (ADHD) and major depressive disorder both involve symptoms regarding concentration difficulties [10], and any instrument measuring the full symptomatology of any of these constructs will inevitably tap into the other construct. Further, overlap may occur when an instrument is applied to measure a mental health construct containing items that are identically worded, or similar to, criteria used to diagnose the population studied, and when results – despite this overlap – are reported as a separate mental health construct. For example, the Strengths and Difficulties Questionnaire (SDQ; [17]), assesses emotional and behavioural problems in four specific scales (emotional symptoms, conduct problems, hyperactivity, and peer relationship problems). Notably, the SDQ can be used to screen for specific NDD, such as ASD and ADHD (e.g., [18, 19]), and to measure emotional and behavioural problems more broadly in children with different NDD (e.g., [20–22]). The specific scales can be combined to form the broad-band scales of internalising (emotional symptoms and peer relationships problems) and externalising problems (conduct problems and hyperactivity) and a total difficulties scale (the sum of all four). Each specific scale consists of five items, and in the case of the peer relationship problems scale, several items are either closely related to or overlapping diagnostic criteria for ASD (e.g., “Rather solitary, tends to play alone”). Whether this overlap is problematic or not is related to which scales are reported and how the results are interpreted. For example, when the score of the peer relationships problems is reported as an indication of ASD the overlap is necessary (e.g., [18]) and when subscales with overlap are purposely omitted (e.g., [23, 24]) the risk for bias due to overlap is avoided. However, when the broad-band scales or the total scale are reported as an indication of a mental health problem construct there is a risk that disability-related difficulties are confused with mental health problems. This could lead to inflated scores which could contribute to incorrect conclusions about differences between groups.

The second domain covered in the review concerns bias arising from an over-reliance on a single type of informant in reporting the mental health problems in focus. Evidence shows that correlations between different types of informants (e.g., parents, teachers, children) can be low to modest depending on the combination [25–27]. Low inter-informant correlation does not necessarily mean that one informant is right and the other one is wrong or implies measurement error [28]. More likely, it relates to systematic differences among informants, such as the contexts in which they observe behaviour [29]. For example, a child may display hyperactivity in school but not at home. Relying on a single informant might provide an incomplete picture of mental health problems. A multi-informant approach is often advised to reduce bias when studying child mental health issues [26, 30]. However, the number and types of informants needed to provide a valid representation may depend on factors like the child’s age, as suggested by the larger discrepancy between informants seen in older as compared to younger children [25].

The third risk of bias domain, which can be considered a special case of the second domain, involves the exclusion of the child’s perspective, and the use of a parent as a sole informant. Exclusion of the child’s perspective might lead to bias through the depression-distortion hypothesis, i.e., the tendency for depressed mothers to exaggerate descriptions of child problems [31]. This risk is highlighted separately in this review because some aspects of mental health problems are inherently subjective. This subjectivity could be an explanation for the larger informant discrepancies observed in internalising problems, such as anxiety and depression, as compared to externalising problems [25–27]. Furthermore, there is a strong ethical argument for including the child’s perspective whenever possible. According to the United Nations Convention on the Rights of the Child [32], every child has a right to be heard in matters that concern the child. However, it is not realistic to expect all children to be able to self-report mental health problems. For children with NDD, the disability itself may be associated with problems with self-reporting, for example, difficulties with interpreting questions, retrieving information, and generating responses, related to underlying cognitive processes such as long-term memory, working memory, and judgment [33, 34]. Some children with NDD, such as those with severe-profound ID, by definition, have a level of impairment in cognitive and communicative functions [10], which makes the use of self-rating questionnaires improbable [35]. Similarly, young children may not have developed the necessary level of cognitive functioning to self-rate on mental health problems, regardless of NDD or not (see for example [36, 37]). However, determining the specific age and cognitive level at which children’s self-report reaches acceptable validity is challenging. For example, Varni, Limbers, and Burwinkle [38] demonstrated that typically developing children as young as five years may be able to make valid reports on their health-related quality of life using the Pediatric Quality of Life Inventory™ (PedsQL™). However, a later analysis of PedsQL™ data showed insufficient psychometric properties for many children between the ages of five and seven years [39]. For children with mild-moderate ID, some evidence indicates that self-rating of mental health problems may be feasible from 11 years, using standard self-rating instruments such as the SDQ [40] or the Youth Self-Report [41], with some adaptations made to the procedure (i.e., questions administered as an interview [40, 41], allowing item content to be explained [41]). Any effort to identify a specific and universal age and cognitive functioning threshold for child self-ratings is likely to fail since validity is also influenced by material and procedural factors [42]. Still, if a child’s self-report is not sought, when possible, an important perspective on the mental health problem is missing. Importantly, this is not the same as saying that there is no merit to parent reports, but rather that parent and child reports are not interchangeable.

The fourth and final risk of bias domain concerns the appropriateness of instruments used to measure mental health problems in the population. Many scales, such as the SDQ and the Child Behavior Checklist (CBCL; [43, 44]), were originally developed for typically developing children. Using these scales could be problematic if manifestations of mental health problems differ between typically developing children and those with NDD. For example, it has been argued that the number and type of symptoms for some psychiatric disorders need to be adapted for use with people with ID [45]. This would imply that questions in diagnostic interviews and screening questionnaires need to be phrased differently. Additionally, all questionnaires and interview procedures, presume some level of cognitive and communicative functioning in respondents. A cognitively accessible design reduces cognitive demands and supports cognitive processes to enable respondents to interpret and respond to assessment items as intended [46]. For example, in many scales, the respondent is to consider a time frame of several weeks or months when rating items. A valid response to the items in such scales presumes a comparatively high level of episodic memory functioning in the respondent, which should pose a bigger challenge to children with impairments in memory functions, such as those with ID [47], than children without memory impairments. Self-assessment could be made a feasible option for a larger proportion of children with NDD through the development of more accessible instruments. This could be achieved by adapting well-established scales to the needs of children with NDD (e.g., [48]) or developing new scales suitable for the target group (e.g., [49]). Of course, not all scales need to be changed to be valid for use in specific groups of children with NDD, as demonstrated by the tentative evidence concerning the use of SDQ and YSL in children with mild-moderate ID [40, 41]. But in samples where cognitive impairments vary or are unknown, higher cognitive accessibility should increase the likelihood of valid responses. This is also true for parent-rated versions of scales. In ID [50], for example, there is a strong genetic component, indicating that cognitive support needs are likely to be expected in many parents of children with ID as well as the children themselves.

In summary, the four domains (see Table 1) involve known methodological challenges that researchers and clinicians attempting to track the longitudinal change in mental health problems in children with NDD need to consider and manage. Common risk of bias appraisal tools like the checklists used in the Critical Appraisal Skills Programme [8] do not provide detailed instructions for assessing bias in these specific yet important domains. Given that many other design features need to be considered when assessing the risk of bias, there is a possibility that these NDD-specific questions may be overlooked. Hence, the purpose of this study was (1) to develop an approach to assessing the risk of bias associated with the four identified domains and (2) to assess the risk of these biases in a recently conducted systematic review of longitudinal trajectories of mental health problems in children with NDD [7]. The research questions for this study were:

Is the risk for overlap between the outcome measures and the criteria used to define the study group (i.e., the NDD) dealt with satisfactorily?
To what extent has a multi-informant approach been taken to capture variation in mental health problem expression in different contexts?
(a) Is the child’s perspective represented in the assessment of the mental health problem? (b) When not, is there a reasonable basis for excluding child self-assessment?
Are the instruments and procedures designed to be cognitively accessible, or have they been adapted in some way to the specific needs of the study group?

**Table 1:** Four domains presenting challenges in measuring longitudinal changes in mental health problems in children with neurodevelopmental disorders (NDD)

Domain	Description
1	Conceptual overlap between mental health problems and NDD diagnostic criteria
2	Over-reliance on a single informant/perspective
3	Unwarranted omission of the child’s perspective
4,	The use of instruments not designed for or adapted to children with NDD

The present study builds on findings from an earlier systematic review of longitudinal mental health problem trajectories in children with NDD [7]. That review identified indications of methodological issues that were not adequately captured by the standard risk of bias tool employed. To better understand the scope and nature of these issues, additional data extraction from the included studies was required. By addressing these four key questions, this study sought to contribute to a better understanding of the methodological weaknesses and strengths of the field of mental health problem trajectories in children with NDD. The findings will be summarised and used to critically evaluate how the field has dealt with the challenges posed.

The study protocol for the systematic review of mental health problem trajectories in children with NDD [7] was registered in PROSPERO (142,412). Some aspects of the design, e.g., search strategy and eligibility criteria, are summarised or appended as supplementary material in the current review, but a more comprehensive description can be found in Danielsson et al. [7]. Taken together, the reviews adhere to the Preferred Reporting Items for Systematic Review and Meta-Analysis (PRISMA) guidelines [51].

### Search strategy

Searches were performed in PsycINFO, ERIC, Web of Science, PubMed, and CINAHL in September 2019 and June 2021 with combinations of words (i.e., synonyms, examples, or MeSH-terms) representing the constructs “mental health”, “disability”, “longitudinal”, and “child”. The searches resulted in 94,662 records, which were reduced to 72,582 with duplicates removed. Another 8,599 were identified by going through the reference lists of relevant reviews. The records were then screened in a three-stage process based on title, abstract, and full-length texts. Due to the large number of identified records, 22 reviewers were involved in the process. An overview of the flow of records through the study is provided in Supplementary Fig. 1, and the detailed eligibility criteria are outlined in Supplementary Table 1.

### Selection criteria

Studies were included based on the eligibility criteria found in Supplementary Table 1. In short, longitudinal studies of mental health problems (defined broadly) in children under 19 years of age with NDD were included. Studies with at least three waves of data collection (with two or more years between the first and last wave) were included. Studies, where the mental health problems of interest were the explicit target of an intervention, were excluded, along with papers written in any language other than English. No limitations regarding publication year were applied.

### Data extraction process

For the original review [7], the extraction of relevant information was done independently by random pairs of reviewers (*n* = 22) and synthesised by a third reviewer. In cases where the original reviewers disagreed, the third did an independent extraction of data and made a final decision based on all three sources. For the current study, key variables relating to measurement constructs/concepts, respondents, and cognitive accessibility were added, and the additional data for these variables were extracted by one investigator (M.I.). In ambiguous cases, another investigator (H.D. or C.I.) was consulted and a consensus decision was made. Information about diagnostic criteria and instruments was retrieved from relevant diagnostic manuals like the DSM-5 and instrument manuals. Most of the data reported were extracted from the included studies for the current study. However, some of the study characteristics (e.g., study populations’ mean age at the first data point), are included in the present study for context, even though they have been reported previously [7].

### Risk of bias assessment protocol

The general risk of bias assessment of the included studies has been previously reported by Danielsson et al. [7]. For the current study, the first author (M.I.), developed a supplementary tool to assess the risk of bias in four domains addressing specific challenges related to the measurement of longitudinal trajectories of mental health problems in children with NDD. Specific assessment criteria (questions) were formulated for each domain to guide the evaluation of the risk of bias. The risk of bias for each domain was rated on a three-point scale based on the responses to the underlying questions (Table 2 provides a detailed description of the assessment for each domain): “Low” (indicating minimal risk), “High” (indicating elevated risk), and “Unclear” (reflecting uncertainties in the bias assessment, either due to mixed findings or insufficient information). The overall risk of bias, across the four domains, was also assessed for each study according to the following principles: (1) if any level of risk of bias (“low”, “unclear”, or “high”) was assigned more frequently than any of the others in the four domains then the overall risk of bias was rated at that level, (2) if “high” and “low” was assigned two times each the overall risk of bias was set to “unclear”, and (3) if two domains were considered to have a “high” or “low” risk of bias and the other two an “unclear”, the overall risk of bias was described as “unclear”. It is important to underscore that this supplement is not intended as a standalone instrument to assess the total risk of bias for a study, but as a complement to standard instruments, for example, the Cochrane Collaboration’s tool [52] or the Critical Appraisal Skills Programme checklist for cohort studies [8].

**Table 2:** A supplementary risk of bias tool for studies assessing mental health problems in children with NDD

Domains	Conceptualisation and rationale	Response options
1) Bias due to overlap between the outcome studied and core characteristics of the study group	Articles were examined for information on whether the authors were aware of any overlap between the dependent and independent variables and if it was somehow dealt with in the analysis and/or interpretation of results	Low = no overlaps between any of the instruments and diagnostic criteria of populations studied (positive answer to 1a) or all overlaps addressed (positive answer to 1b); Unclear = overlaps discussed but not fully addressed (can’t tell on 1b) or overlap could not be assessed (can’t tell on 1a); High = at least one overlap identified that was not mentioned (negative answer to 1a and 1b)
1a) Are the mental health problems studied separated from the core characteristics of the disability of the participating children?	The items of all applied instruments were compared to the most recently published diagnostic criteria for the study groups in search of potential item-criteria overlaps. An overlap was indicated when the item and the diagnostic criteria had a) verbatim similarity, b) a synonymic relationship (e.g., hyperactive and overactive), or c) when either the item or the diagnostic criteria could be considered a more concrete example of the other (e.g., difficulties with social interaction and difficulties in turn-taking). The reason for choosing the most recent diagnostic manual instead of manuals contemporary to the included studies was that later versions have incorporated new scientific evidence on what constitutes core aspects of different diagnoses	Yes = no overlaps between the items in the instrument and diagnostic criteria identified; No = one or more overlaps identified; Can’t tell = diagnostic criteria and/or items needed for comparison not available or overlap for other reasons not possible to assess
1b) Were the conceptual overlaps addressed in the article?	Data on any mention of overlap and strategies to deal with the overlap were extracted	Yes = all overlaps addressed through design or statistical elements; No = no mention of overlap or mention of some reported scores with conceptual overlap but not others; Can’t tell = overlaps mentioned but not fully addressed
2) Bias due to insufficient recruitment of different types of informants (i.e., perspectives and contexts) reporting on the studied mental health problem(s)	A pragmatic criterion for “insufficient” was used: for children going to school (defined as 6 years or older during some part of the follow-up period), two different types of informants were considered sufficient	Low = two or more different types of informants rating the same outcome in at least one case or any number of informants if the children were below 6 years of age at all waves of data collection; Unclear = information on informants’ or participants’ age lacking; High = one informant in studies with children aged 6 or more at one data point or more
2a) How many different informants reported on the child’s mental health problems?	The number of informants representing different contexts (school, home, etc.) or perspectives (parent proxy rating, researcher observation, etc.) for each reported mental health problem outcome was extracted	Numerical
2b) Which informants reported on the child’s mental health problems?	Data on the type of informants were extracted	Parent = mother, father, primary caregiver; Teacher = preschool- or schoolteacher; Child = self-rated by the child; Observation = observed directly by a health professional or member of the research team
2c) Were the participants under six years of age at all data collection points?	The mean age of participants was extracted (or estimated based on the interval between data points if not reported) for each data point	Yes = mean age less than 6 at all data points; No = mean age 6 or above on at least one data point; Can’t tell = no information or not possible to estimate mean age for one or more data points
3) Bias due to the unwarranted omission of the child’s perspective in the measurement(s) of mental health problem(s)	Data on whether the outcome measures were self-reported when possible was extracted	Low = self-report was not deemed possible (negative answer to 3a) or the children rated their mental health problems on at least one mental health problem outcome; Unclear = not possible to say if child rating would have been possible or no information on whether the mental health problems were child rated (can’t tell on 3a or 3b); High = no child-reported data (negative answer to 3b), even though child report was deemed feasible (positive answer to 3a)
3a) Was it theoretically plausible to gather information on mental health problems directly from the participating children at a majority of the follow-ups?	The evaluation of whether self-rating was theoretically plausible was made based on the age and estimated level of intellectual functioning of the participants. A rather conservative limit (9 years) for self-rating in children without major cognitive deficits was applied. The reason for this was that many of the children without an intellectual disability may still have specific cognitive deficits that could interfere with self-rating to some extent. The age limit for children with cognitive deficits equivalent to mild intellectual disability (11 years) was based on earlier studies indicating that self-rating is possible from that age without specific adaptations [40, 41]. Children with more severe levels of cognitive deficits were not considered capable of self-reporting in the current review, although that could certainly be debated	Yes = the participating children were age 9 or older (for groups where a majority did not have an intellectual disability or IQ < 70) or 11 (for children with an intellectual impairment equivalent to mild intellectual disability or IQ 55–70 measured with a test of intellectual functioning or an acceptable proxy) at half or more of the waves of data collection points; No = the participating children were younger than 9 years of age (no intellectual disability or mean IQ > 70 for a majority of participants) or 11 (intellectual functioning equivalent to mild intellectual disability for a majority) at most waves or were children with more severe intellectual impairments of any age; Can’t tell = no data on the age and/or level of intellectual functioning of participants presented
3b) Did the children rate their mental health problems?	Data on whether the outcome measures were self-reported or not were extracted	Yes = at least one of the mental health problem outcomes was self-rated; No = none of the mental health problem outcomes were self-rated; Can’t tell = not possible to tell if the mental health problem outcomes were child-rated or not
4) Bias due to the use of instruments and procedures that were not designed to measure mental health problems in children with NDD	Articles were examined for any mentions of adaptations aiming at increasing the cognitive accessibility of the instruments applied to measure the mental health problem trajectories (e.g., the use of visual support, allowing the interviewer to explain words that the respondent did not comprehend, or providing additional examples) and/or whether these instruments were specifically developed for use in children with NDD	Low = all instruments applied in the study were either adapted to the population or designed for use in the population (positive answer to 4a or 4b); Unclear = some of the applied instruments were adapted to or designed for the population or not possible to extract information; High = instruments were not adapted to or designed for use in the population (negative answer to 4a or 4b)
4a) Were the instruments and procedures intended to be used as a measure of mental health problems in children with NDD?	Data were extracted on whether it was explicitly stated that the instruments were developed to be used with children with NDD	Yes = explicitly stated that at least one of the instruments used to measure mental health problems was developed to be used with children with NDD; No = instrument originally developed for use with typically developing children; Can’t tell = not possible to extract information on the originally intended target group for the instruments
4b) Were the instruments and procedures adapted to the study group in any way?	Data were extracted on whether adaptations of material or procedure aiming at lowering cognitive demands or in any other way adapting them to children with NDD were explicitly mentioned	Yes = one or more adaptations were mentioned in the article; No = no adaptations were mentioned or there were adaptions made but for other reasons; Can’t tell = not possible to determine if adaptations were made or not

### Data analysis

Analyses were carried out in R [53] with RStudio [54]. The R packages *papaja* [55] and *robvis* [56] were used to compile the manuscript and to make the risk of bias figures. The data extracted from the included studies and a reproducible version of the manuscript is available at https://osf.io/hjrqc/.

## Results

A total of 49 original studies were identified through the screening and eligibility process and included in the current review (see Table 3 for an overview of the included studies). Of these, at least 18 reported data from participants that, based on the name of the project or resemblances in participant characteristics, were likely to also have been participants in one or more of the other included studies. Disregarding this overlap, this review includes data from 9,446 participating children. The participants’ mean age ranged from 0.51 to 12.30 years at the first wave of data collection and 4.50 to 23.20 years at the last. The mean number of data collection waves was 5 (range 3 to 17) and the mean total length of follow-up was 5.62 years (range 2.00 to 16.74 years). A total of 148 scale scores were identified when counting the scales reported on the most general level in each included study, i.e., specific subscales were only counted in the absence of a reported total scale score or broad-band scale score. The scores were derived from 34 different instruments.

**Table 3:** Characteristics of the included studies

	Participants				Follow-up	
Study	Diagnosis	Age (y)	n	Females (%)	Length (y)	Occasions (n)
Alsem 2013 [57]	CP	2.50	92	41.30	2	3
Anderson 2011 [58]	ASD	9.75	65	10.77	9	17
Auerbach 2008 [59]	Dyscalculia	11.10	29	51.72	6	3
	Dyscalculia-NP	11.10	29	51.72	6	3
Baribeau 2021 [60]	ASD	3.34	421	15.44	7	8
Biederman 1996 [61]	ADHD	10.60	128	0.00	4	3
Ciciolla 2014 [62]	Delay	3.00	110	32.73	2	3
Colvert 2021 [63]	ASD	4.00	135	13.33	9	3
Cornish 2012 [64]	Fragile X	8.17	48	0.00	2	3
Fielding-Gebhardt 2020 [65]	Fragile X	9.13	55	20.00	7	3
Flouri 2015 [66]	ASD	3.13	165	21.82	4	3
	ASD + ADHD	3.11	44	6.82	4	3
Gotham 2015 [67]	DD	12.30	56	39.00	7	7
Green 2005 [68]	DD	3.90	13	23.08	3	6
Harvey 2015 [69]	ADHD	3.68	75	38.67	3	4
Hauser-Cram 2016 [70]	DD	3.00	169	46.15	15	5
Hickey 2020 [71]	ASD	9.07	159	13.21	NR	3
Hogan 2014 [72]	Hearing	4.75	93	NR	6	4
Holmbeck 2010 [73]	Spina bifida	8.34	68	45.59	6	4
Horbach 2020 [74]	SLD	6.21	27	33.33	5	5
	SLD + ADHD	6.21	15	26.67	5	5
	ADHD	6.21	13	30.77	5	5
Hoza 2010 [75]	ADHD	9.97	513	20.27	6	4
Hunsche 2020 [76]	ASD	7.70	178	17.42	3	4
Kates 2019 [77]	22q11.2DS	11.87	87	47.13	9	4
Lahey 2016 [78]	ADHD	5.24	125	14.40	12	13
		9.24	125	14.40	8	9
		10.24	125	14.40	7	8
Li 2020 [79]	ASD	4.56	59	0.00	2	3
Lindsay 2007 [80]	SSLD	8.25	69	24.64	4	3
Midouhas 2013 [81]	ASD	3.00	209	16.75	4	3
Moskowitz 2020 [82]	Fragile X	6.71	153	18.95	NR	5
Mrug 2012 [83]	ADHD	10.35	300	20.00	6	3
Murray-Close 2010 [84]	ADHD	10.00	536	18.66	6	4
Musser 2016 [85]	ADHD	9.53	388	30.93	2	3
Peverill 2019 [86]	ASD	3.41	396	15.66	3	4
Rai 2018 [87]	ASD	10.00	96	17.71	8	6
Rosema 2015 [88]	TBI mild	5.19	13	46.15	16	5
	TBI moderate	4.89	40	70.00	17	5
	TBI severe	5.09	22	63.64	17	5
Sigafoos 2000 [89]	DD	3.90	13	23.08	3	6
Sipal 2010 [90]	CP	11.23	110	36.36	3	4
St Clair 2011 [91]	SLI	7.00	234	23.50	9	4
Steinhausen 2003 [92]	ADHD	10.20	35	17.14	3	3
Stringer 2020 [93]	ASD	11.60	158	10.13	12	3
Tan 2014 [94]	CP 1–4 y	1.50	97	44.33	3	4
	CP 5–8 y	6.25	116	34.48	2	3
	CP 9–15 y	11.00	108	37.04	3	4
Vaillancourt 2017 [95]	ASD	3.19	392	15.56	3	4
Van keer 2021 [96]	SDD	3.10	25	68.00	2	3
Vaughn 1993 [97]	LD	6.00	10	40.00	3	4
Vaughn 1994 [98]	LD	6.00	10	40.00	5	5
Wall 2019 [99]	Fragile X	0.51	116	25.00	4	8
Wei 2014 [100]	LD	11.59	722	38.37	3	3
	LD + ADHD	11.63	303	20.13	3	3
	ADHD + ED	11.17	569	14.94	3	3
Williams 2016 [101]	ADHD	0.70	112	25.00	6	4
	ADHD-S	0.74	648	35.65	6	4
Woodman 2015 [102]	DD	3.00	176	44.89	15	5
Woodruff-Borden 2010 [103]	WS	6.67	45	53.33	4	9
Yeates 2006 [104]	TBI severe	9.90	53	26.42	4	3
	TBI moderate	10.50	56	26.79	4	3
Zendarski 2021 [﻿^105^]	ADHD	10.70	130	0.00	3	3

The reported mean age at the first wave of data collection and the time between the first and last wave were estimated based on information in the article, supplementary material, or another study reporting data from the same group of participants when not specifically reported. Disabilities/diagnoses

*Abbreviations:* *22q11.2DS* 22q11.2 deletion syndrome, *ADHD* Attention-deficit hyperactivity disorder, *ADHD-S* ADHD symptomatic, *ASD* Autism spectrum disorder, *CP* cerebral palsy, *delay* developmental delays, *DD* Developmental disabilities, *ED* Emotional disturbances, *hearing* Hearing problems, *LD* learning disabilities, *Dyscalculia-NP* nonpersistent dyscalculia, *SDD* significant cognitive and motor developmental delay, *SLI* specific language impairment, *SLD* specific learning disorder, *SSLD* specific speech and language difficulty, *TBI* traumatic brain injury, *WS* Williams syndrome. Other *abbreviations:* *NR* not reported

### Risk of bias domain 1: Conceptual overlap

There was some level of overlap between one or more items and diagnostic criteria in 33.8% of the identified outcomes. Table 4 demonstrates examples of overlaps in two of the included studies and how the risk of bias was assessed. An overview of all studies with at least one overlap (44.9% of studies) is displayed in Supplementary Table 2. In studies where an overlap was identified, 77.3% contained at least one outcome with an overlap that was neither addressed nor discussed. The most frequently occurring instrument-diagnosis combination with a conceptual overlap was SDQ and ASD (*n* = 3). ADHD was the diagnosis where diagnostic criteria most commonly overlapped with at least one item in a reported mental health problem outcome (32.0% of the identified overlaps when including both pure ADHD groups and groups with ADHD and co-occurring diagnoses), followed by ASD (24.0%), and developmental disabilities (16.0%). ADHD, ASD, and developmental disabilities accounted for a larger proportion of the studies with overlap (72.0%) than would have been expected by the size of their combined share of the study groups in the included studies (30.4%).

**Table 4:** Examples of conceptual overlap between mental health problem outcomes and diagnostic criteria from two of the included studies

			Overlap					
Study	Group	Scale	Score	Item	Diagnostic criteria	Code	Addressed	Risk of bias
Anderson 2011 [58]	Autism spectrum disorder	Aberrant Behavior Checklist	Lethargy/Social withdrawal subscale	Fixed facial expression(s)	“…a lack of facial expressions…”	ASD A3, DSM-5	Can’t tell	Unclear
			Hyperactivity subscale	Pays no attention when spoken to	“…failure of normal back-and-forth conversation…”	ASD A1, DSM-5	No	High
			Irritability subscale	Cries over minor things	“…extreme distress at small changes…”	ASD A3, DSM-5	No	High
Auerbach 2008 [59]	Dyscalculia	Child Behavior Checklist	Total	Poor school work	“Difficulties learning and using academic skills…”	SLD A, DSM-5	No	High
			Attention problems subscale	Poor school work	“Difficulties learning and using academic skills…”	SLD A, DSM-5	No	High
	Nonpersistent dyscalculia	Child Behavior Checklist	Total	Poor school work	“Difficulties learning and using academic skills…”	SLD A, DSM-5	No	High
			Attention problems subscale	Poor school work	“Difficulties learning and using academic skills…”	SLD A, DSM-5	No	High

Risk of bias in the first domain and one example of an overlap between an item in a scale measuring mental health problems and one of the diagnostic criteria used to define the population for two of the included studies

*Abbreviations* *ASD* Autism spectrum disorder, *DSM* Diagnostic and Statistical Manual of Mental Disorders, *SLD* specific learning disorder

### Risk of bias domain 2: Multi-informant approach

The risk of bias due to the lack of relevant perspectives on the mental health problems outcome was rated as high in 79.6% of the included studies (see Table 5 for examples of how the risk of bias was assessed in this domain and Supplementary Table 3 for an overview of all the included studies). Information about the mental health problem of interest was collected from multiple informants in 12.2% of the studies and relying on one informant was deemed justifiable, due to the young age of the population, in 8.2%.

**Table 5:** Informants recruited in four of the included studies and an assessment of the appropriateness of the informant recruitment approach based on the age of the participants

Study	Scale	Mental health concept	Informants	Age < 6 years	Risk of bias
Alsem 2013 [57]	TNO-AZL Preschool Children Quality of Life Parent Form	Behaviour problems, sleeping problems, and anxiety	Parent	Yes	Low
Anderson 2011 [58]	Aberrant Behavior Checklist	Maladaptive behaviours	Parent	No	High
Auerbach 2008 [59]	Child Behavior Checklist	Behaviour problems	Parent	No	High
Baribeau 2021 [60]	Child Behavior Checklist	Anxiety	Parent	No	High

Risk of bias in the second domain (i.e., lack of a multi-informant approach) and the factors used as the basis for the decision: number of informants reporting on the mental health problem outcomes and if the mean age of the participating children was below six for all data points

*Abbreviation:* *TNO-AZL* Netherlands Organization for Applied Scientific Research/Academic Hospital Leiden Center

### Risk of bias domain 3: Omission of the child’s perspective

The risk of bias due to a lack of the child’s perspective on the child’s mental health problems was rated as high in 24.5% of studies (see Table 6 for examples of how the risk of bias was assessed in this domain and Supplementary Table 4 for an overview of all the included studies). The child’s perspective was missing in 87.8% of the studies, and of these, child self-rating was deemed theoretically feasible in 53.5% based on a combination of participant age and reported level of intellectual functioning.

**Table 6:** Assessment of the feasibility of including the child’s perspective on the mental health problems outcomes in four of the included studies

				Was child rating feasible?		
Study	Groups	Scales	Child rating	Feasible	Factors influencing assessment	Risk of bias
Alsem 2013 [57]	Cerebral palsy	TNO-AZL Preschool Children Quality of Life Parent Form	No	No	No data on IQ or ID-status, < 9 yo on all waves	Low
Anderson 2011 [58]	Autism spectrum disorder	Aberrant Behavior Checklist	No	No	Nonverbal IQ M = 53, > 11 yo on a majority of waves	Low
Auerbach 2008 [59]	Dyscalculia, Nonpersistent dyscalculia	Child Behavior Checklist	No	Yes	IQ M = 99.1/99.4, > 9 yo on all waves	High
Baribeau 2021 [60]	Autism spectrum disorder	Child Behavior Checklist	No	No	IQ M = 58.0, < 9 yo on a majority of waves	Low

Risk of bias in the third domain (i.e., unwarranted omission of the child’s perspective) and the factors used as the basis for the decision: if any of the longitudinal mental health problem outcomes were child-rated and if child rating would have been feasible given the age and intellectual functioning of the participants

*Abbreviations:* *IQ* Intelligence quotient, *TNO-AZL* Netherlands Organization for Applied Scientific Research/Academic Hospital Leiden Center, *yo* years old

### Risk of bias domain 4: The use of instruments designed for or adapted to children with NDD

Only 8.8% of the different instruments applied were originally designed for use in children with NDD: the Aberrant Behavior Checklist, the Repetitive Behavior Scale-Revised, and the Scale for Emotional Development-Revised. No study reported that adaptations had been made to any instrument to make them more accessible or in other ways suitable for children with NDD (see Table 7 for examples of how the risk of bias was assessed in this domain and Supplementary Table 5 for an overview of all the included studies).

**Table 7:** Scales measuring mental health problems across four of the included studies and their suitability for use in the studied populations

Study	Scale	Intended population	Designed for	Adapted	Risk of bias
Alsem 2013 [57]	TNO-AZL Preschool Children Quality of Life Parent Form	No	TD	No	High
Anderson 2011 [58]	Aberrant Behavior Checklist	Yes	DD	No	Low
Auerbach 2008 [59]	Child Behavior Checklist	No	TD	No	High
Baribeau 2021 [60]	Child Behavior Checklist	No	TD	No	High

Risk of bias in the fourth domain (i.e., use of instruments originally not intended for use in the NDD population) and the factors used as the basis for the decision: if children with NDD were the intended population and if the scale and/or procedures were somehow adapted to the needs of children with NDD

*Abbreviations:* *DD* Developmental disabilities, *TNO-AZL* Netherlands Organization for Applied Scientific Research/Academic Hospital Leiden Center, *TD* typically developing children

### Overall risk of bias in the four domains

**Fig. 1:**
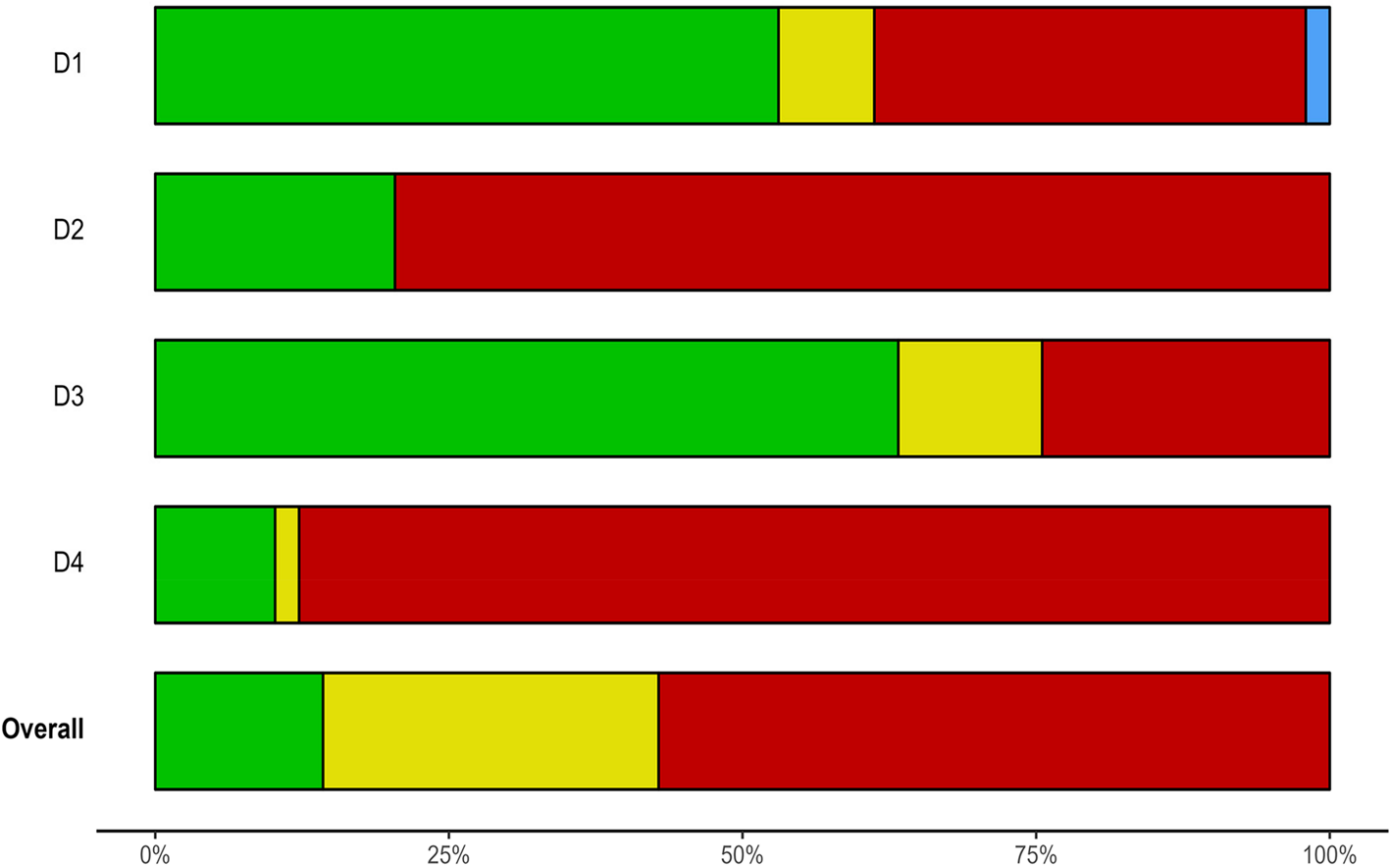
The overall risk of bias for each of the four domains: D1, overlap between mental health problem outcomes and characteristics of the study group; D2, insufficient informants; D3, unwarranted omission of the child’s perspective; and D4, use of instruments not designed for or adapted to the study group (red reflects “high” risk of bias, yellow “unclear”, green “low”, and blue “no information”)

Overall bias across domains was rated as high in 57.1%, unclear in 28.6%, and low in 14.3% of the 49 included studies (see Fig. 1). The domain with the highest proportion of studies rated as having a high risk was the fourth domain, i.e., bias due to the use of instruments not developed for or adapted to children with NDD (high risk of bias in 87.8% of the studies) and the third domain had the fewest risks, i.e., the unwarranted omission of the child’s perspective (high risk of bias in 24.5% of the included studies). All but one [96] of the included studies had a high risk of bias in one or more of the four domains (Supplementary Fig. 2 displays the risk of bias at the individual study level).

## Discussion

The purpose of the present study was to develop and test an approach for assessing the risk of bias in four domains that are of particular importance in longitudinal studies of mental health problems in children with NDD and to assess how common these problems are in the field. Most notably, the results showed that some degree of bias related to these measurement domains was present in almost all of the included studies. Of these four domains, the most frequent concern was the use of instruments not designed for, or adapted to, children with NDD, followed by the restricted number of informants and perspectives represented in the mental health problem data. The risk of bias due to a lack of the child’s perspective and/or overlap between outcomes and the diagnostic criteria used to define the study group was lower compared to the other two domains but was still a substantial issue for the field as a whole.

The results show that conceptual overlap, i.e., mental health problems not being clearly distinguished from NDD diagnostic criteria, is a common concern in studies with populations with symptom-based diagnoses, such as ADHD and ASD, and to a lesser extent in those where diagnoses are based on etiology, such as pediatric traumatic brain injury or Fragile-X. In terms of outcomes, the broad-band internalising and externalising, or total scale scores of SDQ and the CBCL family of scales are often involved in cases with overlap. These scales were developed to screen broadly for problematic levels of emotional and behavioural difficulties in typically developing children and young people and, as such, do not differentiate between symptoms relating to NDD and other disorders or emotional problems on the broad-band levels. There are however subscales, such as the emotional problems scale of SDQ, where the risk for overlap is much lower than the internalising broad-band scale, which also encompasses the peer problems subscale with items very closely related to some NDDs.

The conceptual overlap is, when present, often not discussed and/or adjusted for in the analyses or through other design elements. When addressed, the approach to deal with it spans from mentioning the overlap as a limitation in the discussion [95] to clearly stating that scales with substantial overlap should be interpreted as NDD-related difficulties rather than additional mental health problems [63] and running analyses with and without items with an overlap to get a picture of their influence on the results [90]. The consequence of the conceptual overlap in the field is two-fold. Firstly, it could mean that the levels of mental health problems (as something separated from difficulties relating to NDD) are exaggerated in some groups of children with NDD. Secondly, it makes it hard to tell if a longitudinal change in the measured outcomes reflects changes in NDD-related difficulties or a separate mental health problem. For example, the natural course of NDD-specific difficulties, such as the tendency of a decreasing rate of hyperactivity over time in childhood ADHD [78, 106], risks distorting a mental health problems trajectory if the scale used includes items related to hyperactivity.

A clear majority of studies reported data from only one informant, most frequently a parent, despite recurrent recommendations to apply a multi-informant approach when assessing mental health in children [26, 30]. However, there were exceptions, such as Lahey et al. [78], in which three perspectives (child, teacher, and parent) on the mental health problems being investigated were combined. A restricted number of perspectives in a single study could be less of a problem if the field as a whole had a reasonable distribution of different perspectives. However, as seen in the results, there is an over-reliance on parents in the field today. Direct observations in the children’s natural contexts by researchers were not applied in any of the studies. This reliance on parent-reported data risks under-reporting of behaviours more typically displayed in other contexts than at home, such as problems between peers. Another risk is that parent ratings may be influenced by the parent’s mental health status [31]. This could be especially problematic for parents of children with NDD since they often report symptoms of depression, poor sleep quality, and stress [107]. A specific challenge in studies with a longitudinal design is that the most valid combination of methods and informants changes over time as the child develops. As argued by Rosema et al. [88], parents of younger children are likely to have more knowledge about their child’s mental health problems than parents of adolescents. One possible solution to this dilemma, demonstrated by Lahey et al. [78], is to add, rather than replace, informants as the child grows older.

The child’s perspective was missing in about a quarter of studies where child self-rating was deemed theoretically possible based on the participant’s age and level of intellectual functioning. Since some aspects of mental health problems are intrinsically covert (subjective), and difficult to measure without having the child describe their mental health (as pointed out by Woodruff-Borden et al. [103]), omitting the child’s perspective risks leading to a skewed picture with an overemphasis on overt behaviours. In the long run, this could lead to a self-fulfilling prophecy, where externalising problems are more often included as outcomes than internalising problems based on results from earlier studies. If the unwarranted omission of the child’s perspective is unevenly distributed between studies involving children with NDD and typical development, it follows that it could be difficult to disentangle real differences in profiles of emotional and behavioural differences between the groups from artifacts stemming from the methodological differences.

No examples of self-rating instruments specifically adapted to or designed to be cognitively accessible were identified in the current review. Very few of the scales were explicitly developed for use in the NDD population. Some instruments were claimed to have adequate psychometric properties when used in children with NDD but it was beyond the scope of the current review to go through all evidence on the psychometric properties of the included scales when used in the NDD population and evaluate the validity of such claims. Still, the use of instruments not developed for the population targeted in a study can lead to problems conceptually and practically. For example, mental health problems may have atypical presentations in children with NDD [45, 108], which means that questions may need to be phrased differently than with typically developing children. Further, when self-report is sought, scales need to be carefully designed to optimise cognitive accessibility.

## Limitations

The validity and generalisability of the results of the present review are influenced by a few limitations that need to be discussed. First, even though the definitions and operationalisations applied were well-grounded, it should be noted that there may be reasons for considering other specific thresholds which would have led to slightly different outcomes. For example, a rather conservative definition of mental health problem-NDD overlap was applied, in that only explicit overlaps between diagnostic criteria and items in scales were considered. However, many etiology-based diagnoses are also closely linked to specific behavioural profiles, e.g., Fragile-X with ID and ASD [109]. Widening the definition of overlap to include difficulties typically associated with a disability would have resulted in more overlap being identified. At the same time, such a definition would have led to difficulties in drawing a clear line between NDD-related difficulties and common co-existing difficulties. Second, the present review did not quantify the extent of conceptual overlap in each of the included studies and therefore does not give a full picture of the risk of bias in that domain. Some of the studies used scales with hundreds of items whereof only a few overlapped diagnostic criteria, while other scales were much shorter and had more items with overlap. Future research will have to further investigate the exact extent of the problem. Third, the most frequently used risk of bias level across the four domains was used when calculating the overall risk of bias across domains, rather than generalising the highest risk of bias seen in a specific domain to the overall level. The reason for choosing this approach was that it allowed important variability to be exposed overall: generalising from the highest-rated item would have risked all included articles deemed to have the same (high) level of bias overall. Finally, the systematic search for evidence on which this study is based was conducted in 2021, hence more recent publications may have addressed these potential risks of bias more fully. However, the main aim of the current study was to develop and try out an approach for assessing these specific risks of bias rather than summarising the most recent evidence in the field of longitudinal mental health problem trajectories in children with NDD.

## Clinical implications and future research

The overarching recommendation emanating from the results is that methods for collecting information on mental health problems in children with NDD could be chosen with more consideration than appears to have been done to date to avoid a partly skewed picture of mental health problems in both clinical and research settings. Several steps need to be taken to reduce the risk of bias in future studies. When selecting which scale(s) to use, it is important to:

1. Choose conceptually clear scales and subscales.
2. Prioritise self-report over parent-report, especially for internalising problems. When considering self-report, it is important to acknowledge that factors other than child-related factors determine whether it is feasible or not. The cognitive accessibility of scales is equally important and should be considered along with validity when deciding between scales.
3. As with typically developing children, a multi-informant approach is recommended, especially but not only for older children who spend a large part of the day at school, with peers, or in other contexts without parents. In longitudinal studies where the first data collecting point takes place in early childhood, it is advised that self-report, teacher-report, and/or direct observations are added as the child grows older. Future studies need to investigate the barriers to applying a multi-informant approach in longitudinal studies of mental health problems in children with NDD.
4. When no valid and accessible scale exists for a specific construct, a primary focus ought to be to develop one or adapt a scale originally developed for typically developing children to fit the needs of children with NDD. In research, more attention needs to be directed toward the challenge of developing and validating cognitively accessible self-report scales and procedures to assess mental health problems.

Finally, it is recommended that the four domains addressed in the current review should be considered whenever assessing the risk of bias in studies of mental health problems, and related constructs, in children with NDD in future systematic reviews. Without consideration of these additional potential risks of bias in this type of study, we may overestimate the quality of the evidence available.

## Conclusions

The present study aimed to develop an approach for critically reviewing four measurement-related domains in studies investigating longitudinal trajectories of mental health problems in children with NDD, as well as to assess the risk of bias in these domains within the literature. All but one of the included studies had a high level of risk of bias in one or more domains, most commonly (1) the use of instruments not designed for or adapted to children with NDD, and in descending order, (2) an insufficient number of informants and perspectives represented in the mental health problem data, (3) overlap between the mental health problem outcomes and the diagnostic criteria used to define the study group, and (4) a lack of the child’s perspective. Taken together, these risks of bias could lead to a skewed picture of the mental health problems of children with NDD, through processes leading to both over- (e.g., conceptual overlap) and underestimation (e.g., use of instruments not developed for children with NDD). To minimise these problems in future research and clinical contexts, it is advised that instruments and procedures are chosen following a few guiding principles. Researchers and clinicians should seek to include multiple perspectives on the mental health issue of interest, use scales without conceptual overlap, preferably developed for children with NDD, and wherever possible in the form of cognitively accessible self-report scales. If no such scales exist, the development and validation of new scales should be a priority.

## Supplementary Information

Supplementary Material 1.

## Data Availability

The data that was extracted from the included studies and a reproducible version of the manuscript, including the code, are available at https://osf.io/hjrqc/.
