# Supplementary material for: Measurement issues in longitudinal studies of mental health problems in children with neurodevelopmental disorders
Source: BMC Psychol. 2025 Mar 18;13:267. doi: 10.1186/s40359-025-02450-4 (PMC11917076; doi:10.1186/s40359-025-02450-4)
Supplement: Supplementary file 1 — Supplementary Material 1. [file 40359_2025_2450_MOESM1_ESM.pdf]

Supplementary Material: Measurement Issues in Longitudinal Studies of Mental Health Problems in Children With Neurodevelopmental Disabilities

Magnus Ivarsson, Henrik Danielsson, & Christine Imms

This document contains material supplementary to the article entitled “Measurement Issues in Longitudinal Studies of Mental Health Problems in Children With Neurodevelopmental Disabilities”, which was published in BMC Psychology.

Table of Contents

Supplementary Table 1 Eligibility criteria for the screening process **2**

Supplementary Table 2 Conceptual overlap between mental health problem outcomes and diagnostic criteria **3**

Supplementary Table 3 Informants recruited in the included studies and an assessment of the appropriateness of the informant recruitment approach based on the age of the participants **13**

Supplementary Table 4 Assessment of the feasibility of including the child’s perspective on the mental health problems outcomes in the included studies **19**

Supplementary Table 5 Scales measuring mental health problems across studies and their suitability for use in the studied populations **26**

Supplementary Fig. 1 PRISMA flow diagram depicting how records were identified, screened, and assessed for eligibility at different phases **31**

Supplementary Fig. 2 Risk of bias for each of the four domains, i.e., overlap between mental health problem outcomes and characteristics of the study group (D1), insufficient informants (D2), unwarranted omission of the child perspective (D3), use of instruments not designed for or adapted to the study group (D4), as well as the overall risk of bias assessment for the included studies **32**

Supplementary Table 1 Eligibility criteria for the screening process

Aspects considered	Inclusion	Exclusion
Participants	Studies reporting data separately for one or more groups of participants aged 18 years or below (for at least 2 of the time points), with one or more of the following disabilities: intellectual disability, autism spectrum disorder, attention deficit hyperactivity disorder, hearing impairment, seeing impairment, cerebral palsy, acquired brain injury, communication disorders, specific learning disorders, motor disorders [including developmental coordination disorder, tic, Tourette] or another diagnosed disability primarily associated with impairment(s) in the ICF-domains seeing and related functions (b210-b229), hearing functions (b230), mental functions (b1) and/or neuromusculoskeletal and movement-related functions (b7).	Studies reporting data from a study group with one or more participants older than 18 years of age and/or typical development and/or condition or disability other than the ones listed in the inclusion criteria.
Intervention(s)	Intervention studies aiming at other outcomes than mental health and where it is reasonable to suggest that mental health will not be affected by intervention.	Any aiming at changing mental health
Method	Studies with longitudinal data series with at least 3 time points (where the mean time between the first and last time point is 2 years or longer) with the same measure of mental health problems across time points.	Cross-sectional studies, longitudinal studies with less than 3 time points
Main outcome(s)	Mental health problems defined broadly (e.g., anxiety, behavior problems, depression, sleep problems) and conceptualized as a categorical or continuous variable	Any other
Language	English	Any other
Publication year	Any	NA

Supplementary Table 2 Conceptual overlap between mental health problem outcomes and diagnostic criteria

			Overlap					
Study	Group	Scale	Score	Item	Diagnostic criteria	Code	Addressed	Risk of bias
Anderson 2011	Autism spectrum disorder	Aberrant Behavior Checklist	Lethargy/Social withdrawal subscale	Fixed facial expression(s)	"…a lack of facial expressions…"	ASD A3, DSM-5	Can't tell	Unclear
			Hyperactivity subscale	Pays no attention when spoken to	"…failure of normal back-and-forth conversation…"	ASD A1, DSM-5	No	High
			Irritability subscale	Cries over minor things	"…extreme distress at small changes…"	ASD A3, DSM-5	No	High
Auerbach 2008	Dyscalculia	Child Behavior Checklist	Total	Poor school work	"Difficulties learning and using academic skills…"	SLD A, DSM-5	No	High
			Attention problems subscale	Poor school work	"Difficulties learning and using academic skills…"	SLD A, DSM-5	No	High
	Nonpersistent dyscalculia	Child Behavior Checklist	Total	Poor school work	"Difficulties learning and using academic skills…"	SLD A, DSM-5	No	High

Study	Group	Scale	Overlap: Score	Item	Diagnostic criteria	Code	Addressed	Risk of bias
			Attention problems subscale	Poor school work	"Difficulties learning and using academic skills…"	SLD A, DSM-5	No	High
Ciciolla 2014	Developmental delays	Child Behavior Checklist	Internalising broad-band scale	Acts too young for age	"…fails to meet expected developmental milestones in several areas of intellectual functioning…"	Global Developmental Delay, DSM-5	No	High
			Externalising broad-band scale	Can't concentrate, can't pay attention for long	"…fails to meet expected developmental milestones in several areas of intellectual functioning…"	Global Developmental Delay, DSM-5	No	High
Colvert 2021	Aautism spectrum disorder	Strengths and Difficulties Questionnaire	Peer problems subscale	Rather solitary, tends to play alone	"…absence of interest in peers…"	ASD A3, DSM-5	Yes	Unclear
			Pro-social behaviour subscale	Considerate of other people's feelings	"…reduced sharing of interests, emotions, or affect…"	ASD A1, DSM-5	Yes	Unclear

			Overlap					
Study	Group	Scale	Score	Item	Diagnostic criteria	Code	Addressed	Risk of bias
Flouri 2015	Aautism spectrum disorder	Strengths and Difficulties Questionnaire	Peer problems subscale	Rather solitary, tends to play alone	"…absence of interest in peers…"	ASD A3, DSM-5	No	High
	Aautism spectrum disorder+Attention-deficit hyperactivity disorder	Strengths and Difficulties Questionnaire	Peer problems subscale	Rather solitary, tends to play alone	"…absence of interest in peers…"	ASD A3, DSM-5	No	High
Gotham 2015	Developmental disabilities	Child Behavior Checklist/Aberrant Behavior Checklist	Anxiety subscale	Clings to adults or too dependent (Child Behavior Checklist)	"…failure to meet developmental and sociocultural standards for personal independence…"	ID B, DSM-5	Can't tell	Unclear
			Affective/depressive subscales	Has trouble making decisions (Adult Behavior Checklist)	"Deficits in intellectual functions, such as reasoning, problem solving, planning, abstract thinking…"	ID A, DSM-5	Can't tell	Unclear
Green 2005	Developmental disabilities	Aberrant Behavior Checklist	Total	Abnormal repetitive movements	"Stereotype or repetitive motor movements…"	ASD B1, DSM-5	No	High
Hauser-Cram 2016	Developmental disabilities	Child Behavior Checklist	Internalising broad-band scale	Acts too young for age	"…failure to meet developmental	ID A, DSM-5	No	High

Study	Group	Scale	Overlap: Score	Overlap: Item	Overlap: Diagnostic criteria	Overlap: Code	Overlap: Addressed	Risk of bias
					[…] standards…”			
			Externalising broad-band scale	Can’t concentrate, can’t pay attention for long	“Deficits in intellectual functions…”	ID A, DSM-5	No	High
Hickey 2020	Aautism spectrum disorder	Teacher Report Form	Total	Would rather be alone than with others	“…absence of interest in peers.”	ASD A3, DSM-5	No	High
Horbach 2020	Attention-deficit hyperactivity disorder	Child Behavior Checklist	Total	Breaks rules at home, school, or elsewhere	“Often leaves seat in situations when remaining seated is expected”	ADHD A2b, DSM-5	No	High
			Externalising broad-band scale	Breaks rules at home, school, or elsewhere	“Often leaves seat in situations when remaining seated is expected”	ADHD A2b, DSM-5	No	High
			Attention problems subscale	Can’t concentrate, can’t pay attention for long	“Often has difficulty sustaining attention…”	ADHD A1b, DSM-5	No	High
	Specific learning disorder	Child Behavior Checklist	Total	Poor school work	“The affected academic skills […] cause significant interference with academic […]	SLD B1, DSM-5	No	High

			Overlap					
Study	Group	Scale	Score	Item	Diagnostic criteria	Code	Addressed	Risk of bias
					performance…”			
			Attention problems subscale	Poor school work	“The affected academic skills […] cause significant interference with academic […] performance…”	SLD B1, DSM-5	No	High
	Specific learning disorder+Attention-deficit hyperactivity disorder	Child Behavior Checklist	Total	Breaks rules at home, school, or elsewhere	“Often leaves seat in situations when remaining seated is expected”	ADHD A2b, DSM-5	No	High
			Externalising broad-band scale	Breaks rules at home, school, or elsewhere	“Often leaves seat in situations when remaining seated is expected”	ADHD A2b, DSM-5	No	High
			Attention problems subscale	Can’t concentrate, can’t pay attention for long	“Often has difficulty sustaining attention…”	ADHD A1b, DSM-5	No	High
Hoza 2010	Attention-deficit hyperactivity disorder	Children’s Depression Inventory	Total	I have to push myself all the time to do my schoolwork	…has difficulty remaining focused during lectures…	ADHD A1b, DSM-5	No	High

			Overlap					
Study	Group	Scale	Score	Item	Diagnostic criteria	Code	Addressed	Risk of bias
Midouhas 2013	Aautism spectrum disorder	Strengths and Difficulties Questionnaire	Total difficulties	Rather solitary, tends to play alone	"…absence of interest in peers…"	ASD A3, DSM-5	No	High
Mrug 2012	Attention-deficit hyperactivity disorder	Children's Depression Inventory/Beck Depression Inventory	Average item	I have to push myself all the time to do my schoolwork (Children's Depression Inventory)	"…has difficulty remaining focused during lectures…"	ADHD A1b, DSM-5	No	High
		Multidimensional Anxiety Scale for Children/Beck Anxiety Inventory	Average item	I feel restless and on edge (Multidimensional Anxiety Scale for Children)	"Often runs about or climbs in situations where it is inappropriate. (Note: In adolescents or adults, may be limited to feeling restless)."	ADHD A2c, DSM-5	No	High
Peverill 2019	Aautism spectrum disorder	Child Behavior Checklist	Internalising broad-band scale	Avoids looking others in the eye	"…abnormalities in eye contact…"	ASD A2, DSM-5	No	High
		Repetitive Behavior Scale-Revised	Total	Resists changing activities; difficulty with transitions	"…difficulties with transitions…"	ASD B2, DSM-5	Can't tell	Unclear

			Overlap					
Study	Group	Scale	Score	Item	Diagnostic criteria	Code	Addressed	Risk of bias
Sigafoos 2000	Developmental disabilities	Aberrant Behavior Checklist	Total	Abnormal repetitive movements	"Stereotype or repetitive motor movements…"	ASD B1, DSM-5	No	High
Sipal 2010	Cerebral palsy	Child Behavior Checklist	Total	Poorly coordinated or clumsy	"Abnormal gross and fine motor functioning and organization (reflecting abnormal motor control) are the core features of CF."	Cerebral Palsy Clarification 6, Rosenbaum et al. (2007, p. 10)	Yes	Low
			Social problems subscale	Speech problem	"…motor problems can lead to difficulties with […] articulation of speech…"	Cerebral Palsy Clarification 6, Rosenbaum et al. (2007, p. 10)	Yes	Low
			Attention problems subscale	Stares blankly	"Vision, hearing and other sensory modalities may be affected…"	Cerebral Palsy Clarification 15, Rosenbaum et al. (2007, p. 10)	Yes	Low
Steinhausen 2003	Attention-deficit hyperactivity disorder	Child Behavior Checklist	Total	Can't sit still, restless, or hyperactive	"A persistent pattern of […] hyperactivity-	ADHD A, DSM-5	Yes	Unclear

			Overlap					
Study	Group	Scale	Score	Item	Diagnostic criteria	Code	Addressed	Risk of bias
					impulsivity”			
			Externalising broad-band scale	Talks too much	“Often talks excessively”	ADHD A2f, DSM-5	Yes	Unclear
			Attention problems subscale	Can’t sit still, restless, or hyperactive	“A persistent pattern of […] hyperactivity-impulsivity”	ADHD A, DSM-5	Yes	Unclear
			Aggressive behaviour subscale	Talks too much	“Often talks excessively”	ADHD A2f, DSM-5	Yes	Unclear
Vaillancourt 2017	Aautism spectrum disorder	Child Behavior Checklist	Internalising broad-band scale	Doesn’t answer when people talk to him/her	“…failure of normal back-and-forth conversation…”	ASD A1, DSM-5	Can’t tell	Unclear
Vaughn 1993	Learning disabilities	Revised Behavior Problem Checklist	Attention problems subscale	School work is messy, sloppy	“Difficulties learning and using academic skills…”	SLD A, DSM-5	No	High
			Anxious-withdrawn subscale	Feels he or she can’t succeed	“Even with an array of appropriate accommodations or services at home, at school, or in the workplace, the	SLD Severe specifier, DSM-5	No	High

			Overlap					
Study	Group	Scale	Score	Item	Diagnostic criteria	Code	Addressed	Risk of bias
					individual may not be able to complete all activities efficiently…”			
Vaughn 1994	Learning disabilities	Revised Behavior Problem Checklist	Attention problems subscale	School work is messy, sloppy	“Difficulties learning and using academic skills…”	SLD A, DSM-5	No	High
			Anxious-withdrawn subscale	Feels he or she can’t succeed	“Even with an array of appropriate accommodations or services at home, at school, or in the workplace, the individual may not be able to complete all activities efficiently…”	SLD Severe specifier, DSM-5	No	High
Wei 2014	Attention-deficit hyperactivity disorder+Emotional disturbances	Social Skills Rating Systems	Problem behaviour subscale	Is easily distracted	“Is often easily distracted by extraneous stimuli”	ADHD A1h, DSM-5	No	High
	Learning disabilities	Social Skills	Problem behaviour	Is easily distracted	“Is often easily distracted by	ADHD A1h,	No	High

			Overlap					
Study	Group	Scale	Score	Item	Diagnostic criteria	Code	Addressed	Risk of bias
	+Attention-deficit hyperactivity disorder	Rating Systems	subscale		extraneous stimuli”	DSM-5		
Woodman 2015	Developmental disabilities	Child Behavior Checklist	Internalising broad-band scale	Acts too young for age	“…failure to meet developmental […] standards…”	ID A, DSM-5	No	High
			Externalising broad-band scale	Can’t concentrate, can’t pay attention for long	“Deficits in intellectual functions…”	ID A, DSM-5	No	High

Note. Risk of bias in the first domain and one example of an overlap between an item in a scale measuring mental health problems and one of the diagnostic criteria used to define the population. Studies with no clear conceptual overlap are excluded from the table. One of the included studies [57] could not be assessed for overlap due to an inability to retrieve the scale applied in the study. For another study [58], the assessment was based on the instrument used at the last data collection wave (ABC) as the investigator was not able to access the scales applied in earlier waves. Because the different scales were treated as equivalent in the analyses, relying on the ABC in the assessment of overlap was deemed acceptable. Abbreviations used in the table: attention-deficit hyperactivity disorder (ADHD), autism spectrum disorder (ASD), Diagnostic and Statistical Manual of Mental Disorders (DSM), intellectual disability (ID), specific learning disorder (SLD).

Supplementary Table 3 Informants recruited in the included studies and an assessment of the appropriateness of the informant recruitment approach based on the age of the participants

Study	Scale	Mental health concept	Informants	Age <6 years	Risk of bias
Alsem 2013	TNO-AZL Preschool Children Quality of Life Parent Form	Behaviour problems, sleeping problems, and anxiety	Parent	Yes	Low
Anderson 2011	Aberrant Behavior Checklist	Maladaptive behaviours	Parent	No	High
Auerbach 2008	Child Behavior Checklist	Behaviour problems	Parent	No	High
Baribeau 2021	Child Behavior Checklist	Anxiety	Parent	No	High
Biederman 1996	Kiddie Schedule for Affective Disorders and Schizophrenia—Epidemiological	Psychiatric disorders	Parent, child	No	Low
Ciciolla 2014	Child Behavior Checklist	Internalising and externalising symptoms	Parent	Yes	Low
Colvert 2021	Strengths and Difficulties Questionnaire	Emotional and behavioural difficulties	Parent	No	High
Cornish 2012	Conners Teacher Rating Scales—Revised	Oppositional behaviour problems	Teacher	No	High
Fielding-Gebhardt 2020	Child Behavior Checklist	Challenging behaviours	Parent	No	High
Flouri 2015	Strengths and Difficulties Questionnaire	Emotional and conduct problems	Parent	No	High
Gotham 2015	Child Behavior Checklist/Adult Behavior Checklist	Anxiety	Parent	No	High

Study	Scale	Mental health concept	Informants	Age <6 years	Risk of bias
		Depressive symptoms	Parent	No	High
Green 2005	Aberrant Behavior Checklist	Aberrant behaviour	Teacher	No	High
Harvey 2015	Disruptive Behavior Rating Scale	Oppositional defiant disorder symptoms	Parent	No	High
Hauser-Cram 2016	Child Behavior Checklist	Behaviour problems	Parent	No	High
Hickey 2020	Teacher Report Form	Emotional and behavioural problems	Teacher	No	High
Hogan 2014	Strengths and Difficulties Questionnaire	Psychosocial outcomes	Parent, teacher	No	Low
Holmbeck 2010	Children's Depression Inventory	Internalising symptoms	Child	No	High
Horbach 2020	Child Behavior Checklist	Emotional and behavioural problems	Parent	No	High
Hoza 2010	Children's Depression Inventory	Depressive symptoms	Child	No	High
	DSM-IV Conduct Disorder Checklist	Aggressive behaviour	Parent	No	High
Hunsche 2020	Child Behavior Checklist	Suicidal ideation and self-injurious behaviour	Parent	No	High
Kates 2019	Kiddie Schedule for Affective Disorders and Schizophrenia for School-Age Children—Present and Lifetime Version/Structured Clinical Interview for DSM-IV-TR axis I Disorders	Psychiatric diagnoses	Parent, child	No	Low

Study	Scale	Mental health concept	Informants	Age <6 years	Risk of bias
Lahey 2016	Diagnostic Interview Schedule for Children	Depression symptoms	Parent, child	No	Low
		Anxiety symptoms	Parent, child	No	Low
	Diagnostic Interview Schedule for Children/Disruptive Behavior Disorders Rating Scale—DSM-IV Version	Conduct disorder symptoms	Parent, teacher	No	Low
	Diagnostic Interview Schedule for Children	Conduct disorder symptoms	Child	No	Low
	Diagnostic Interview Schedule for Children/Disruptive Behavior Disorders Rating Scale—DSM-IV Version	Oppositional defiant disorder symptoms	Parent, teacher	No	Low
Li 2020	Early Childhood Inventory—4	Internalising and externalising problems	Parent	No	High
	Emotion Expression Questionnaire	Negative emotion expression	Parent	No	High
Lindsay 2007	Strengths and Difficulties Questionnaire	Behavioural, emotional, and social difficulties	Parent, teacher	No	Low
Midouhas 2013	Strengths and Difficulties Questionnaire	Psychopatohology	Parent	No	High
Moskowitz 2020	Repetitive Behavior Scale—Revised	Self injurous and compulsive behaviour	Parent	No	High
Mrug 2012	Children's Depression Inventory/Beck Depression Inventory	Depression	Child	No	High

Study	Scale	Mental health concept	Informants	Age <6 years	Risk of bias
	Multidimensional Anxiety Scale for Children/Beck Anxiety Inventory	Anxiety	Child	No	High
Murray-Close 2010	DSM-IV Conduct Disorder Checklist	Aggressive and antisocial behaviour	Parent	No	High
Musser 2016	Kiddie Schedule for Affective Disorders and Schizophrenia—Epidemiological Oppositional Defiant Disorder module	Oppositional defiant disorder symptoms	Parent	No	Low
	DSM-based checklist	Oppositional defiant disorder symptoms	Teacher	No	Low
Peverill 2019	Child Behavior Checklist	Externalising and internalising behaviour	Parent	No	High
	Repetitive Behavior Scale—Revised	Restricted and repetitive behaviours	Parent	No	High
	Children's Sleep Habits Questionnaire	Sleep problems	Parent	No	High
Rai 2018	Short Mood and Feelings Questionnaire	Depressive symptoms	Child	No	High
Rosema 2015	Personality Inventory for Children—Revised/Behavior Assessment Scale for Children/Adult Behavior Checklist	Internalising and externalising symptoms	Parent	No	High
Sigafoos 2000	Aberrant Behavior Checklist	Aberrant behaviour	Teacher	No	High
Sipal 2010	Child Behavior Checklist	Behaviour problems	Parent	No	High
St Clair 2011	Strengths and Difficulties Questionnaire	Behavioural, emotional, and social difficulties	Teacher	No	High

Study	Scale	Mental health concept	Informants	Age <6 years	Risk of bias
Steinhausen 2003	Child Behavior Checklist	Behavioural change	Parent	No	High
Stringer 2020	Strengths and Difficulties Questionnaire	Mental health symptoms	Parent	No	High
Tan 2014	TNO-AZL Preschool Children Quality of Life Parent Form	Psychological functioning	Parent	Yes	Low
	TNO-AZL Children's Quality of Life Parent Form	Psychological functioning	Parent	No	High
	TNO-AZL Children's Quality of Life Child Form	Psychological functioning	Child	No	High
Vaillancourt 2017	Child Behavior Checklist	Mental health problems	Parent	No	High
Van keer 2021	Scale for Emotional Development-Revised	Social-emotional development	Parent	Yes	Low
Vaughn 1993	Revised Behavior Problem Checklist	Behaviour problems	Teacher	No	High
Vaughn 1994	Revised Behavior Problem Checklist	Behaviour problems	Teacher	No	High
Wall 2019	Infant Behavior Questionnaire—Revised/Early Childhood Behavior Questionnaire/Children's Behavior Questionnaire	Negative affect	Parent	Yes	Low
Wei 2014	Social Skills Rating Systems	Internalising problems, externalising problems and hyperactivity	Teacher	No	High

Study	Scale	Mental health concept	Informants	Age <6 years	Risk of bias
	School records	School behaviour problems	School records	No	High
Williams 2016	Australian Temperament Scale—Short Form, Infant and Child Version	Emotional dysregulation	Parent	No	High
	Single item	Sleep problems	Parent	No	High
Woodman 2015	Child Behavior Checklist	Behaviour problems	Parent	No	High
Woodruff-Borden 2010	Anxiety Disorders Interview Schedule for DSM-IV: Parent Version	Anxiety disorders	Parent	No	High
Yeates 2006	Teacher Report Form	Emotional and behavioural functioning	Teacher	No	High
Zendarski 2021	Strengths and Difficulties Questionnaire	Emotional and conduct problems	Parent	No	High

Note. Risk of bias in the second domain (i.e., lack of a multi-informant approach) and the factors used as the basis for the decision: number of informants reporting on the mental health problem outcomes and if the mean age of the participating children was below six for all data points. Abbreviations used in the table: Diagnostic and Statistical Manual of Mental Disorders (DSM), Netherlands Organization for Applied Scientific Research/Academic Hospital Leiden Center (TNO-AZL).

Supplementary Table 4 Assessment of the feasibility of including the child's perspective on the mental health problems outcomes in the included studies

				Was child rating feasible?		
Study	Groups	Scales	Child rating	Feasible	Factors influencing assessment	Risk of bias
Alsem 2013	Cerebral palsy	TNO-AZL Preschool Children Quality of Life Parent Form	No	No	No data on IQ or ID-status, <9 yo on all waves	Low
Anderson 2011	Autism spectrum disorder	Aberrant Behavior Checklist	No	No	Nonverbal IQ M = 53, >11 yo on a majority of waves	Low
Auerbach 2008	Dyscalculia, Nonpersistent dyscalculia	Child Behavior Checklist	No	Yes	IQ M = 99.1/99.4, >9 yo on all waves	High
Baribeau 2021	Autism spectrum disorder	Child Behavior Checklist	No	No	IQ M = 58.0, <9 yo on a majority of waves	Low
Biederman 1996	Attention-deficit hyperactivity disorder	Kiddie Schedule for Affective Disorders and Schizophrenia—Epidemiological	Yes	Yes	Included some degree of child self-assessment	Low
Ciciolla 2014	Developmental delays	Child Behavior Checklist	No	No	Child developmental status M = 60.0, <9 yo on a majority of waves	Low
Colvert 2021	Autism spectrum disorder	Strengths and Difficulties Questionnaire	No	No	IQ M = 92, <9 yo on a majority of waves	Low
Cornish 2012	Fragile X	Conners Teacher Rating Scales—Revised	No	No	IQ M = 52.0-65.5, <11 yo on all waves.	Low

Study	Groups	Scales	Child rating	Was child rating feasible?		Risk of bias
				Feasible	Factors influencing assessment	
Fielding-Gebhardt 2020	Fragile X	Child Behavior Checklist	No	Can't tell	No data on IQ or ID-status, >9 yo on all waves	Unclear
Flouri 2015	Autism spectrum disorder, Autism spectrum disorder+Attention-deficit hyperactivity disorder	Strengths and Difficulties Questionnaire	No	No	No data on IQ or ID-status, <9 yo on a majority of waves	Low
Gotham 2015	Developmental disabilities	Child Behavior Checklist/Adult Behavior Checklist	No	Yes	Verbal IQ M = 79.6, >11 yo on all waves	High
Green 2005	Developmental disabilities	Aberrant Behavior Checklist	No	No	No data on IQ or ID-status, <9 yo on a majority of waves	Low
Harvey 2015	Attention-deficit hyperactivity disorder	Disruptive Behavior Rating Scale	No	No	No data on IQ or ID-status, <9 yo on a majority of waves	Low
Hauser-Cram 2016	Developmental disabilities	Child Behavior Checklist	No	Can't tell	ID in 57% but level not specified, <11 yo on a majority of waves	Low
Hickey 2020	Autism spectrum disorder	Teacher Report Form	No	Yes	ID in 35.2%, >9 yo on all waves	High
Hogan 2014	Hearing problems	Strengths and Difficulties Questionnaire	No	No	No data on IQ or ID-status, <9 yo on a majority of waves	Low

				Was child rating feasible?		
Study	Groups	Scales	Child rating	Feasible	Factors influencing assessment	Risk of bias
Holmbeck 2010	Spina bifida	Children's Depression Inventory	Yes	Yes	Included some degree of child self-assessment	Low
Horbach 2020	Specific learning disorder, Attention-deficit hyperactivity disorder	Child Behavior Checklist	No	No	IQ M = 101.55, <9 yo on a majority of waves	Low
Hoza 2010	Attention-deficit hyperactivity disorder	Children's Depression Inventory, DSM-IV Conduct Disorder Checklist	Yes	Yes	Included some degree of child self-assessment	Low
Hunsche 2020	Autism spectrum disorder	Child Behavior Checklist	No	Yes	IQ M >70, >9 yo on a majority of waves	High
Kates 2019	22q11.2 deletion syndrome	Kiddie Schedule for Affective Disorders and Schizophrenia for School-Age Children—Present and Lifetime Version/Structured Clinical Interview for DSM-IV-TR axis I Disorders	Yes	Yes	Included some degree of child self-assessment	Low
Lahey 2016	Attention-deficit hyperactivity disorder	Diagnostic Interview Schedule for Children, Disruptive Behavior Disorders Rating Scale—DSM-IV Version	Yes	Yes	Included some degree of child self-assessment	Low
Li 2020	Autism spectrum disorder	Early Childhood Inventory—4, Emotion Expression Questionnaire	No	No	IQ M = 100.50, <9 yo on a majority of waves	Low

Study	Groups	Scales	Child rating	Was child rating feasible? Feasible	Factors influencing assessment	Risk of bias
Lindsay 2007	Specific speech and language difficulty	Strengths and Difficulties Questionnaire	No	Yes	Non-verbal ability "in the normal range", >9 yo on a majority of waves	High
Midouhas 2013	Autism spectrum disorder	Strengths and Difficulties Questionnaire	No	No	No data on IQ or ID-status, <9 yo on a majority of waves	Low
Moskowitz 2020	Fragile X	Repetitive Behavior Scale—Revised	No	No	IQ M = 56.70, <9 yo on a majority of waves	Low
Mrug 2012	Attention-deficit hyperactivity disorder	Children's Depression Inventory/Beck Depression Inventory, Multidimensional Anxiety Scale for Children/Beck Anxiety Inventory	Yes	Yes	Included some degree of child self-assessment	Low
Murray-Close 2010	Attention-deficit hyperactivity disorder	DSM-IV Conduct Disorder Checklist	No	Yes	IQ M = 100.9 (entire MTA group), >9 yo on all waves	High
Musser 2016	Attention-deficit hyperactivity disorder	Kiddie Schedule for Affective Disorders and Schizophrenia—Epidemiological Oppositional Defiant Disorder module, DSM-based checklist	No	Yes	IQ >74, >9 yo on all waves	High
Peverill 2019	Autism spectrum disorder	Child Behavior Checklist, Repetitive Behavior Scale—Revised, Children's Sleep Habits Questionnaire	No	No	Developmental functioning (IQ proxy) M = 57.35, <9 yo on a majority of waves	Low
Rai 2018	Autism spectrum	Short Mood and Feelings	Yes	Yes	Included some degree of child self-	Low

Study	Groups	Scales	Child rating	Was child rating feasible? Feasible	Factors influencing assessment	Risk of bias
	disorder	Questionnaire			assessment	
Rosema 2015	Traumatic brain injury	Personality Inventory for Children—Revised	No	Can't tell	No data on IQ or ID-status, >9 yo on a majority of waves	Unclear
Sigafoos 2000	Developmental disabilities	Aberrant Behavior Checklist	No	No	No data on IQ or ID-status, <9 yo on a majority of waves	Low
Sipal 2010	Cerebral palsy	Child Behavior Checklist	No	Can't tell	No data on IQ or ID-status, >9 yo on all waves	Unclear
St Clair 2011	Specific language impairment	Strengths and Difficulties Questionnaire	No	Yes	Performance IQ M = 105.98, >9 yo on 50% of waves	High
Steinhausen 2003	Attention-deficit hyperactivity disorder	Child Behavior Checklist	No	Yes	IQ M = 98.8, >9 yo for 50% of waves	High
Stringer 2020	Autism spectrum disorder	Strengths and Difficulties Questionnaire	No	Yes	IQ M = 72.2, >11 yo on all waves	High
Tan 2014	Cerebral palsy	TNO-AZL Preschool Children Quality of Life Parent Form, TNO-AZL Children's Quality of Life Parent Form, TNO-AZL Children's Quality of Life Child Form	Yes	Yes	Included some degree of child self-assessment	Low
Vaillancourt 2017	Autism spectrum disorder	Child Behavior Checklist	No	No	Developmental level (IQ proxy) M = 56.75-61.48, <9 yo on all waves	Low

Study	Groups	Scales	Child rating	Was child rating feasible?		Risk of bias
				Feasible	Factors influencing assessment	
Van keer 2021	Significant cognitive and motor developmental delay	Scale for Emotional Development-Revised	No	No	"Significant cognitive delay", <9 yo on all waves	Low
Vaughn 1993	Learning disabilities	Revised Behavior Problem Checklist	No	No	IQ M = 92, <9 yo on a majority of waves	Low
Vaughn 1994	Learning disabilities	Revised Behavior Problem Checklist	No	No	IQ M = 92, <9 yo on a majority of waves	Low
Wall 2019	Fragile X	Infant Behavior Questionnaire—Revised/Early Childhood Behavior Questionnaire/Children's Behavior Questionnaire	No	No	Developmental level (IQ proxy) M = 58.21-68.17, <9 yo on a majority of waves	Low
Wei 2014	Learning disabilities, Learning disabilities+Attention-deficit hyperactivity disorder, Attention-deficit hyperactivity disorder+Emotional disturbances	Social Skills Rating Systems, School records	No	Yes	Included children classified as having a learning disability or emotional disturbances (but not ID), >9 yo on all waves	High
Williams 2016	Attention-deficit hyperactivity disorder, Attention-deficit hyperactivity disorder symptomatic	Australian Temperament Scale—Short Form, Infant and Child Version, Single item	No	No	No data on IQ or ID-status, <9 yo on a majority of waves	Low
Woodman 2015	Developmental	Child Behavior Checklist	No	Can't tell	No data on IQ or level of ID, >9 yo on a	Unclear

Study	Groups	Scales	Child rating	Was child rating feasible? Feasible	Factors influencing assessment	Risk of bias
	disabilities				majority of waves	
Woodruff-Borden 2010	Williams syndrome	Anxiety Disorders Interview Schedule for DSM-IV: Parent Version	No	Can't tell	No data on IQ, not possible to determine if the sample was >9 yo for at least 50% of waves	Unclear
Yeates 2006	Traumatic brain injury severe, Traumatic brain injury moderate	Teacher Report Form	No	Can't tell	No data on IQ post-injury, >9 yo on all waves	Unclear
Zendarski 2021	Attention-deficit hyperactivity disorder	Strengths and Difficulties Questionnaire	No	Yes	IQ≥70, >9 yo on all waves	High

Note. Risk of bias in the third domain (i.e., unwarranted omission of the child's perspective) and the factors used as the basis for the decision: if any of the longitudinal mental health problem outcomes were child rated and if child rating would have been feasible given the age and intellectual functioning of the participants. Abbreviations used in the table: Diagnostic and Statistical Manual of Mental Disorders (DSM), intellectual disability (ID), intelligence quotient (IQ), Netherlands Organization for Applied Scientific Research/Academic Hospital Leiden Center (TNO-AZL), years old (yo).

Supplementary Table 5 Scales measuring mental health problems across studies and their suitability for use in the studied populations

Study	Scale	Intended population	Designed for	Adapted	Risk of bias
Alsem 2013	TNO-AZL Preschool Children Quality of Life Parent Form	No	TD	No	High
Anderson 2011	Aberrant Behavior Checklist	Yes	DD	No	Low
Auerbach 2008	Child Behavior Checklist	No	TD	No	High
Baribeau 2021	Child Behavior Checklist	No	TD	No	High
Biederman 1996	Kiddie Schedule for Affective Disorders and Schizophrenia—Epidemiological	No	TD	No	High
Ciciolla 2014	Child Behavior Checklist	No	TD	No	High
Colvert 2021	Strengths and Difficulties Questionnaire	No	TD	No	High
Cornish 2012	Conners Teacher Rating Scales—Revised	No	TD	No	High
Fielding-Gebhardt 2020	Child Behavior Checklist	No	TD	No	High
Flouri 2015	Strengths and Difficulties Questionnaire	No	TD	No	High
Gotham 2015	Child Behavior Checklist/Adult Behavior Checklist	No	TD	No	High
Green 2005	Aberrant Behavior Checklist	Yes	DD	No	Low
Harvey 2015	Disruptive Behavior Rating Scale	No	TD	No	High

Study	Scale	Intended population	Designed for	Adapted	Risk of bias
Hauser-Cram 2016	Child Behavior Checklist	No	TD	No	High
Hickey 2020	Teacher Report Form	No	TD	No	High
Hogan 2014	Strengths and Difficulties Questionnaire	No	TD	No	High
Holmbeck 2010	Children's Depression Inventory	No	TD	No	High
Horbach 2020	Child Behavior Checklist	No	TD	No	High
Hoza 2010	Children's Depression Inventory	No	TD	No	High
	DSM-IV Conduct Disorder Checklist	No	TD	No	High
Hunsche 2020	Child Behavior Checklist	No	TD	No	High
Kates 2019	Kiddie Schedule for Affective Disorders and Schizophrenia for School-Age Children—Present and Lifetime Version/Structured Clinical Interview for DSM-IV-TR axis I Disorders	No	TD	No	High
Lahey 2016	Diagnostic Interview Schedule for Children	No	TD	No	High
	Diagnostic Interview Schedule for Children/Disruptive Behavior Disorders Rating Scale—DSM-IV Version	No	TD	No	High
Li 2020	Early Childhood Inventory—4	No	TD	No	High
	Emotion Expression Questionnaire	No	TD	No	High

Study	Scale	Intended population	Designed for	Adapted	Risk of bias
Lindsay 2007	Strengths and Difficulties Questionnaire	No	TD	No	High
Midouhas 2013	Strengths and Difficulties Questionnaire	No	TD	No	High
Moskowitz 2020	Repetitive Behavior Scale—Revised	Yes	ASD	No	Low
Mrug 2012	Children’s Depression Inventory/Beck Depression Inventory	No	TD	No	High
	Multidimensional Anxiety Scale for Children/Beck Anxiety Inventory	No	TD	No	High
Murray-Close 2010	DSM-IV Conduct Disorder Checklist	No	TD	No	High
Musser 2016	Kiddie Schedule for Affective Disorders and Schizophrenia—Epidemiological Oppositional Defiant Disorder module	No	TD	No	High
	DSM-based checklist	No	TD	No	High
Peverill 2019	Child Behavior Checklist	No	TD	No	High
	Repetitive Behavior Scale—Revised	Yes	ASD	No	Low
	Children’s Sleep Habits Questionnaire	No	TD	No	High
Rai 2018	Short Mood and Feelings Questionnaire	No	TD	No	High
Rosema 2015	Personality Inventory for Children—Revised/Behavior Assessment Scale for Children/Adult Behavior Checklist	No	TD	No	High

Study	Scale	Intended population	Designed for	Adapted	Risk of bias
Sigafoos 2000	Aberrant Behavior Checklist	Yes	DD	No	Low
Sipal 2010	Child Behavior Checklist	No	TD	No	High
St Clair 2011	Strengths and Difficulties Questionnaire	No	TD	No	High
Steinhausen 2003	Child Behavior Checklist	No	TD	No	High
Stringer 2020	Strengths and Difficulties Questionnaire	No	TD	No	High
Tan 2014	TNO-AZL Preschool Children Quality of Life Parent Form	No	TD	No	High
	TNO-AZL Children's Quality of Life Parent Form	No	TD	No	High
	TNO-AZL Children's Quality of Life Child Form	No	TD	No	High
Vaillancourt 2017	Child Behavior Checklist	No	TD	No	High
Van keer 2021	Scale for Emotional Development-Revised	Yes	ID	No	Low
Vaughn 1993	Revised Behavior Problem Checklist	No	TD	No	High
Vaughn 1994	Revised Behavior Problem Checklist	No	TD	No	High
Wall 2019	Infant Behavior Questionnaire—Revised/Early Childhood Behavior Questionnaire/Children's Behavior Questionnaire	No	TD	No	High
Wei 2014	Social Skills Rating Systems	No	TD	No	High

Study	Scale	Intended population	Designed for	Adapted	Risk of bias
	School records	No	TD	No	High
Williams 2016	Australian Temperament Scale—Short Form, Infant and Child Version	No	TD	No	High
	Single item	No	TD	No	High
Woodman 2015	Child Behavior Checklist	No	TD	No	High
Woodruff-Borden 2010	Anxiety Disorders Interview Schedule for DSM-IV: Parent Version	No	TD	No	High
Yeates 2006	Teacher Report Form	No	TD	No	High
Zendarski 2021	Strengths and Difficulties Questionnaire	No	TD	No	High

Note. Risk of bias in the fourth domain (i.e., use of instruments originally not intended for use in the NDD population) and the factors used as the basis for the decision: if children with NDD were the intended population and if the scale and/or procedures were somehow adapted to the needs of children with NDD. Abbreviations used in the table: autism spectrum disorder (ASD), developmental disabilities (DD), Diagnostic and Statistical Manual of Mental Disorders (DSM), Netherlands Organization for Applied Scientific Research/Academic Hospital Leiden Center (TNO-AZL), typically developing children (TD).

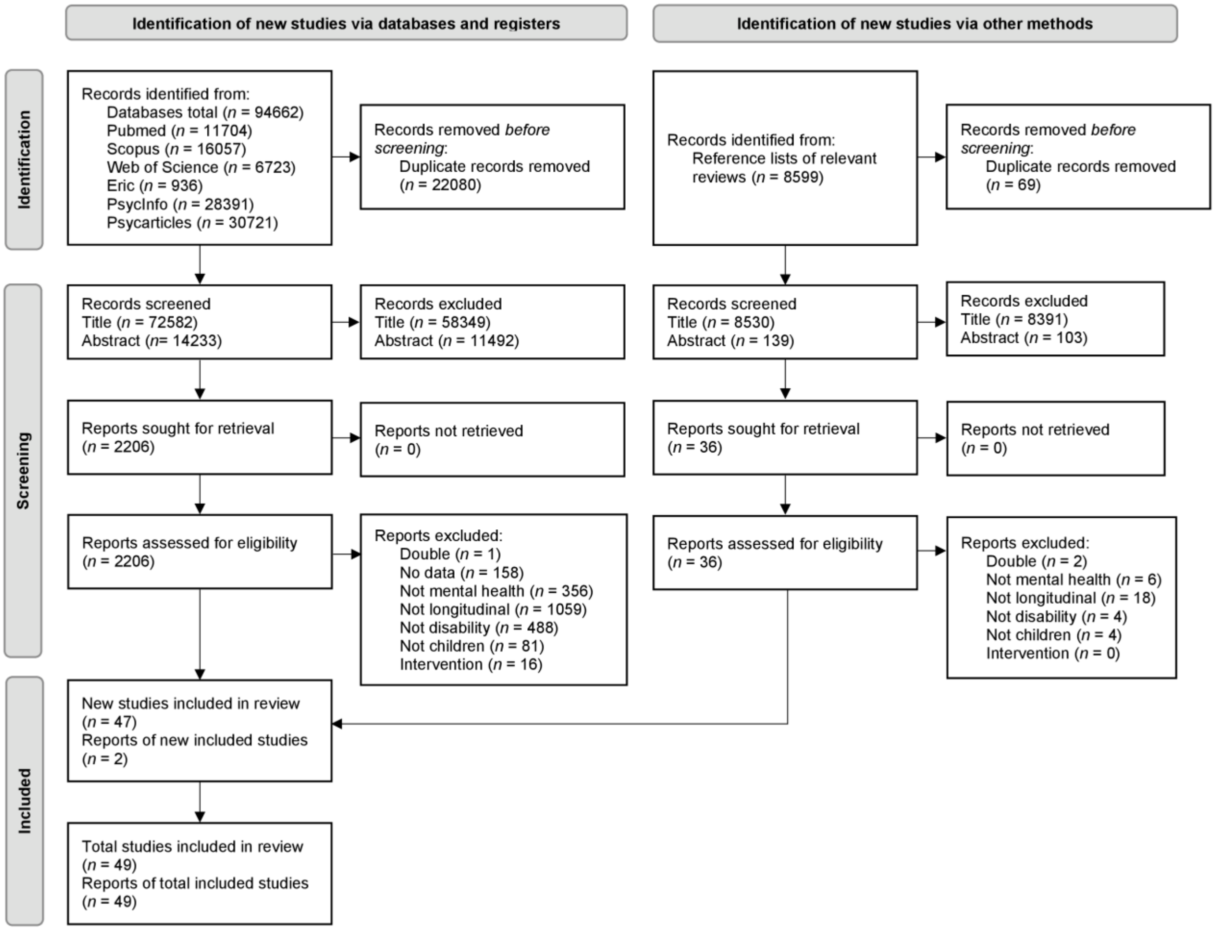

Supplementary Fig. 1 PRISMA flow diagram depicting how records were identified, screened, and assessed for eligibility at different phases

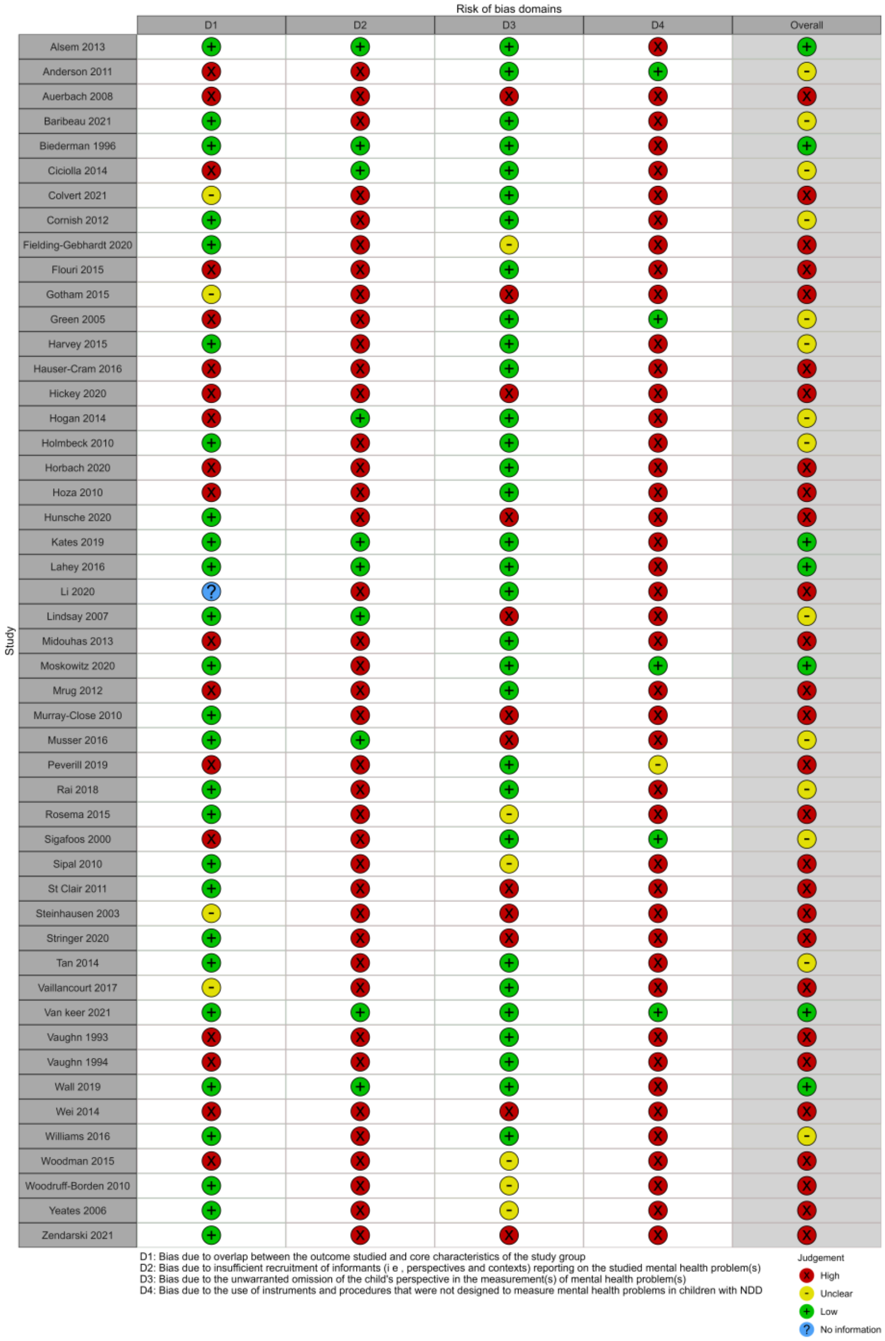

Supplementary Fig. 2 Risk of bias for each of the four domains, i.e., overlap between mental health problem outcomes and characteristics of the study group (D1), insufficient informants (D2), unwarranted omission of the child perspective (D3), use of instruments not designed for or adapted to the study group (D4), as well as the overall risk of bias assessment for the included studies
